# Longitudinal household surveillance for malaria in Rakai, Uganda

**DOI:** 10.1186/s12936-016-1128-6

**Published:** 2016-02-09

**Authors:** Kevin Newell, Valerian Kiggundu, Joseph Ouma, Enos Baghendage, Noah Kiwanuka, Ronald Gray, David Serwadda, Charlotte V. Hobbs, Sara A. Healy, Thomas C. Quinn, Steven J. Reynolds

**Affiliations:** Research Data and Communication Technologies, Inc., Garrett Park, MD USA; Office of HIV/AIDS/Global Health Bureau, USAID Global Health Fellows Program, 1300 Pennsylvania Avenue NW, Washington, 20523 DC USA; Rakai Health Sciences Program, Kalisizo, Uganda; Makerere University Walter Reed Project, Kampala, Uganda; International AIDS Vaccine Initiative (IAVI), Uganda Program, Entebbe, Uganda; Johns Hopkins Bloomberg School of Public Health, Baltimore, MD USA; School of Public Health, Makerere College of Health Sciences, Kampala, Uganda; Batson Children’s Hospital, Division of Infectious Disease, Department of Pediatrics, University of Mississippi Medical Center, Jackson, MS USA; Division of Intramural Research, National Institute of Allergy and Infectious Diseases, National Institutes of Health, Bethesda, MD USA; Johns Hopkins University School of Medicine, Baltimore, MD USA; NIAID/NIH ICER Program, c/o US Embassy Kampala, P.O. Box 7007, Kampala, Uganda

**Keywords:** Malaria, Epidemiology, Surveillance, Household, Children, Rakai, Africa

## Abstract

**Background:**

HIV and malaria exert co-pathogenic effects. Malaria surveillance data are necessary for public health strategies to reduce the burden of disease in high HIV prevalence settings.

**Methods:**

This was a longitudinal cohort study to assess the burden of malaria in rural Rakai, Uganda. Households were visited monthly for 1 year to identify confirmed clinical malaria (CCM), or parasitaemia with temperature >37.5 °C, and asymptomatic parasitaemia (AP). Interviews of the adult or child’s caregiver and clinical and laboratory assessments were conducted. Rapid diagnostic testing for malaria and anaemia was performed if participants were febrile and anti-malarial treatment given per Uganda Ministry of Health 2010 guidelines. Blood was drawn at every household visit to assess for parasitaemia, and blood smears were assessed at the Rakai Health Science Programme laboratory.

**Results:**

A total of 1640 participants were enrolled, including 975 children aged 6 months up to 10 years, 393 adult caregivers, and 272 adolescent/adult household members from 393 randomly selected households in two representative communities. 1459 (89 %) participants completed all study visits. CCM was identified in 304 (19 %) participants, with the highest incidence rate for CCM of 0.38 per person-year (ppy) identified in children <5 years, and rates decreased with age; the rates were 0.27, 0.16, and 0.09 ppy for ages 5–<10 years, 10–<18 years, and adults 18+ years, respectively. AP was identified in 943 (57 %) participants; the incidence rate was 1.99 ppy for <5 years, 2.72 ppy for 5–<10 years, 2.55 ppy for 10–<18 years, and 0.86 ppy among adults, with 92 % of cases being attributed to *Plasmodium falciparum* by smear. 994 (61 %) individuals had at least one positive smear; 342 (21 %) had one positive result, 203 (12 %) had two, 115 (7 %) had three, and 334 (21 %) had >3 positive smears during follow-up. Seasonal rates generally followed the rains and peaked during July, then decreased through November before increasing again.

**Conclusions:**

*Plasmodium falciparum* infection remains high in rural Uganda. Increased malaria control interventions should be prioritized.

*Trial registration* Clinicaltrials.gov identifier NCT01265407

## Background

Malaria is a leading cause of morbidity and mortality in Uganda, accounting for 25–40 % of all outpatient visits at health facilities, 20 % of hospital admissions, and 9–14 % of inpatient deaths [1]. Children under 5 years and pregnant women and HIV-infected individuals bear the greatest burden of disease [2]. Malaria incidence data for this period of time are lacking for most areas of Uganda, particularly in areas of high HIV prevalence, with the most recent Malaria Indicator Surveys having published data collected in 2009 [1] and 2014–2015 [3]. Because HIV and malaria exert co-pathogenic effects [4], malaria surveillance data are necessary for public health strategies to reduce the burden in high HIV prevalence settings. The rural district of Rakai, Uganda, has an HIV prevalence of 9 % (age 15–59 years. cf. national prevalence of 7.3 %) [5]. A study of malaria among children with fever in a hospital setting in the rural Rakai district, Uganda was conducted by this research team and previously described high prevalence of malaria parasitaemia and anaemia among children under 5 years [6]. Because of the continued transmission potential that exists with subclinical parasitaemia, and because many patients do not have ready access to treatment in rural areas, a complementary active-surveillance study was conducted to determine population-based rates of malaria parasitaemia and clinical malaria in Rakai District, and to describe the clinical presentation and prevention strategies being used in households for malaria. This report describes the characteristics of malaria in this rural community with high HIV prevalence [7].

## Methods

Rakai district is on a plateau at an altitude ranging between 750 and 900 m and has fair rainfall throughout the year, with relatively dry periods during January and February and from June through August. Peak rainfall varies from year to year, but occurs typically in March/April and October/November [8]. Malaria is meso- to holo-endemic with year round transmission and highest intensity following the rainy seasons or in communities adjacent to lakes and other mosquito breeding sites. Specifically, the Rakai district has high transmission with estimates preceding this study of more than 100 infective bites per person per year [1]. This was a longitudinal household cohort study, with active-surveillance visits by clinicians every 4 weeks during a 1 year follow-up period for each household. The study period spanned from April 2011–September 2012, which included a 6 month recruitment period of randomly selected households from two communities in Rakai District. The communities and households were identified from the Rakai Community Cohort Study (RCCS) described elsewhere [9].

RCCS household enumeration census data were used to identify households with two or more children between the age of 6 months and 10 years. A statistician from RHSP randomly selected eligible households from the census data. A study nurse approached households on each list sequentially to provide information about the surveillance study to the primary caregiver, and then screened the household for eligibility. The study recruited: the primary caregiver of children from each household; all eligible children aged 6 months up to 10 years; and one additional adult or adolescent participant aged 14 years or older. Due to limited resources the study team was unable to recruit every individual in each household; therefore, one additional adult/adolescent participant was randomly selected among eligible adult/adolescents in the household using the Kish grid method [10]. This recruitment method employs a pre-assigned table of random numbers to identify a respondent within each household unit with the aim to reduce selection bias.

Study visits were made to enrolled households every 4 weeks during a 1 year surveillance period. Visit procedures included: structured interview of the primary caregiver or legal guardian of the children, clinical and laboratory assessment of each participant in the household, and a heel/finger stick for blood smear; slides were read at a later time at the Rakai Health Sciences Program (RHSP) laboratory. If a participant was febrile (>37.5 °C) the study team also performed a rapid diagnostic test and hemoglobin to assess the need to treat for malaria or anemia. Referrals were made for severe malaria to local clinics for care. Anemia was defined as any hemoglobin level <11 g/dl, and further characterized for comparability with the Uganda MIS reports as mild (10–10.9 g/dl), moderate (8–9.9 g/dl), or severe (<8 g/dl). Data collected during the structured interview included self-reported use of malaria prevention methods, including indoor residual spray, mosquito bed net, insecticide-treated bed net and intermittent preventive treatment.

Confirmed clinical malaria (CCM) was defined as evidence of parasitaemia (positive RDT or smear), accompanied by signs/symptoms consistent with malaria, specifically axillary temperature >37.5 °C. Treatment for malaria was provided according to the Ugandan Ministry of Health (MOH) guidelines [11] which include artemisinin combination therapy for uncomplicated malaria. Severe malaria was defined in accordance with the World Health Organization (WHO) guidelines [12], with local modifications due to laboratory constraints, as *Plasmodium falciparum* parasitaemia of any level (or positive RDT) plus one or more of the following: hemoglobin <5 g/dl, prostration, respiratory distress, bleeding, massive haemoglobinuria, recent seizures, coma or obtundation, inability to eat or drink, persistent vomiting or jaundice. Participants identified with suspected severe malaria were referred immediately for in-patient treatment at the nearest health center or hospital.

### Statistical methods

This study is a descriptive, non-comparative study to assess the epidemiology of malaria infection in children aged 6 months–<10 years, and adults living in same households as children. The main objective is to estimate the incidence of asymptomatic and symptomatic malaria in the study population in preparation for future malaria studies in Rakai, including malaria vaccine trials. As data was lacking on malaria incidence in Rakai communities, and on the variance in children aged 6 months–<10 years, sample size calculations were based on a method [13] that applies a pre-determined ratio between the precision width and the standard deviation to estimate sample sizes for descriptive studies using the formula N = 4z^2^S^2^/W^2^. A precision width that is 10 % of the standard deviation is narrow enough to provide a large enough sample size that would adequately describe the incidence of malaria in the study population. Using a 95 % confidence interval (CI) and a ratio of interval width to standard deviation (W/S) of 0.10 requires a total sample size of 1537 participants. A low loss to follow-up rate of 3 % was assumed and by adjusting for a non-response rate of 3 %, the required total sample size was determined to be 1635 [1537/(1–0.06)] participants. The unit of enrolment was the household; therefore, an adequate number of households were enumerated from RCCS census data using known household age cohort structure within these Rakai communities in order to approximate this sample size. Malaria prevalence is reported as proportion of subjects identified during the study period with CCM or AP and stratified by subject characteristics, including age, sex and community. A Chi square statistic was used to compare prevalence across age and gender strata. Incidence estimates are reported per person-year with 95 % CI. A kappa statistic was calculated to measure the level of agreement between RDT and blood smear findings. The malaria attributable fraction (MAF) among febrile subjects is also reported along with 95 % CI for these estimates to provide a measure of the proportion of fevers identified during this study period which can be attributed to malaria. All analyses were conducted using SAS software version 9.4. Copyright, SAS Institute Inc. SAS and all other SAS Institute Inc. product or service names are registered trademarks or trademarks of SAS Institute Inc., Cary, NC, USA.

### Laboratory procedures

Laboratory evaluations included the following at all scheduled surveillance visits: Hemocue^®^ rapid haemoglobin test for febrile subjects and malaria rapid diagnostic test (Malaria Total Quick Test, Cypress Diagnostics, Langdorp, Belgium, sensitivity 99.7 % and specificity 99.5 %.) for febrile subjects; thick and thin slide malaria smears were obtained from all subjects by finger- or heel-prick and evaluated in the RHSP laboratory. The malaria “Total Quick” RDT tests for both antigens specific to *P. falciparum* (HRP2) as well as lactate dehydrogenase, which is an antigen shared by other Plasmodium species that infect humans, including *P. falciparum, Plasmodium ovale, Plasmodium malariae*, and *Plasmodium vivax*. Thick smears were used to identify parasites and quantify parasite count. Thin smears were used for parasite speciation. All slides were read in the RHSP Lab by experienced microscopists who were blinded to the history or signs of clinical malaria. Internal quality assurance of smear reads was conducted by preserving slides and having a separate blinded microscopist reread 10 % of all slides with any discrepant resolved by a third microscopist.

### Ethics

The study was approved by the Uganda Virus Research Institute Science and Ethics Committee, the Uganda National Council for Science and Technology, and the National Institute of Allergy and Infectious Diseases Intramural Institutional Review Board. All study participants at least 18 years of age provided written informed consent prior to screening. Parents or guardians of minors provided written consent and children aged 8 years and older provided assent to the research.

## Results

The primary caregivers in 403 households were visited in their homes and provided information about the study, of which 393 (98 %) agreed to participate. Of the ten households not enrolled, four caregivers refused participation, three caregivers were unable to be contacted, two households had all children away at boarding school, and one household had migrated out of the study area. A total of 1640 participants were enrolled, including 393 (24 %) adult caregivers, 975 (59 %) children <10 years of age, and 272 (17 %) other adult or adolescent members of the households. An average of 2.5 child participants, and an average of 4.2 total participants, were enrolled per household. Table 1 presents a summary of enrolment characteristics.

**Table 1:** Enrollment by community, cohort and gender

	Kalisizo n (%)		Kabira n (%)		Total n (%)
	Male	Female	Male	Female	
Cohort					
Caregivers	07 (2)	202 (51)	05 (1)	179 (46)	393 (24)
Children < 10 years	292 (30)	231 (24)	235 (24)	217 (22)	975 (59)
Additional adult/adolescents	77 (28)	62 (23)	66 (24)	67 (25)	272 (17)
Total	376 (23)	495 (30)	306 (19)	463 (28)	1640

Retention in the annual surveillance study was high with 1459 (89 %) of participants completing the final study visit. Visit adherence was also very high, with only 481 (2.2 %) of 21,618 expected study visits missed; 21,137 (97.8 %) participant visits were completed during the surveillance period. 108 (6.6 %) participants were lost to follow-up, twenty-two (1.3 %) participants withdrew consent, and there were four (0.2 %) deaths. Forty-seven (2.9 %) of participants were terminated due to other circumstances.

There were 21,128 blood smears obtained during the study period. CCM was identified in 304 (19 %) participants (Table 2), with highest proportion of CCM in children <5 years (33 % for males; 25 % for females, p < 0.0001). CCM prevalence decreased with age, with the lowest rate identified among adult females (7 %). The rate of CCM was consistently higher among males than females in all age categories (p < 0.0001). AP was identified in 943 (57 %) participants during the study period. The prevalence of AP was highest in older children (5–<10 years) and adolescents (10–<18 years) and lowest in children under five and adults (18+ years). The prevalence of AP did not differ by gender among children <10 years; however, in older children, adolescents and adults, males had ~10 % higher proportion of AP within each age stratum.

**Table 2:** Prevalence of confirmed clinical malaria and asymptomatic parasitaemia by age and gender

Age (years)	Confirmed clinical malaria n (%)		Asymptomatic parasitaemia n (%)		Total participants n (%)		
	Male	Female	Male	Female	Male	Female	Total
0.5–<5	76 (33)	45 (25)	128 (56)	104 (58)	229 (56)	179 (44)	408 (25)
	121 (30)		232 (57)				
05–<10	68 (23)	52 (19)	196 (66)	169 (63)	298 (53)	269 (47)	567 (35)
	120 (21)		365 (64)				
10–<18	13 (13)	15 (14)	71 (72)	68 (61)	99 (47)	111 (53)	210 (13)
	28 (13)		139 (66)				
18+	7 (12)	28 (7)	31 (55)	176 (44)	56 (12)	399 (88)	455 (28)
	35 (8)		207 (45)				
Total	164 (24)	140 (15)	426 (62)	517 (54)	682 (42)	958 (58)	1640
	304 (19)		943 (57)				

Incidence rate estimates for malaria are presented according to age and gender strata (Table 3). Overall incidence of CCM in this study was 0.23 per person-year (ppy). Rates of CCM follow the same general trend as for prevalence, with youngest children <5 years bearing the greatest burden of malaria (0.38 ppy), and decreasing rates of malaria with increasing age (0.27 ppy in children 5–<10 years, 0.16 ppy in ages 10–<18 years, and 0.09 ppy in adults 18+). Incidence of AP was 1.99 ppy overall, and remained high across all age cohorts: the rate estimate is 1.99 ppy in young children <5 years, 2.72 in children 5–<10 years, 2.55 ppy in ages 10–<18 years, and 0.86 ppy in adults 18+.

**Table 3:** Incidence rate (per person-year) of confirmed clinical malaria and asymptomatic parasitaemia by age and gender

Age (years)	Confirmed clinical malaria (95 % CI)		Asymptomatic parasitaemia (95 % CI)	
	Male	Female	Male	Female
0.5–<5	0.43 (0.34, 0.51)	0.32 (0.23, 0.40)	1.97 (1.64, 2.29)	2.01 (1.58, 2.44)
	0.38 (0.32, 0.44)		1.99 (1.72, 2.25)	
05–<10	0.29 (0.23, 0.35)	0.26 (0.19, 0.33)	2.86 (2.55, 3.17)	2.56 (2.21, 2.91)
	0.27 (0.23, 0.32)		2.72 (2.47, 2.97)	
10–<18	0.19 (0.11, 0.27)	0.13 (0.08, 0.19)	2.70 (2.19, 3.20)	2.43 (1.95, 2.90)
	0.16 (0.12, 0.21)		2.55 (2.20, 2.90)	
18+	0.17 (0.07, 0.28)	0.08 (0.05, 0.11)	1.22 (0.92, 1.52)	0.81 (0.70, 0.93)
	0.09 (0.06, 0.12)		0.86 (0.75, 0.96)	
Total	0.31 (0.27, 0.35)	0.18 (0.15, 0.21)	2.40 (2.19, 2.61)	1.70 (1.54, 1.85)
	0.23 (0.21, 0.26)		1.99 (1.86, 2.11)	

*CI* confidence interval

**Fig. 1:**
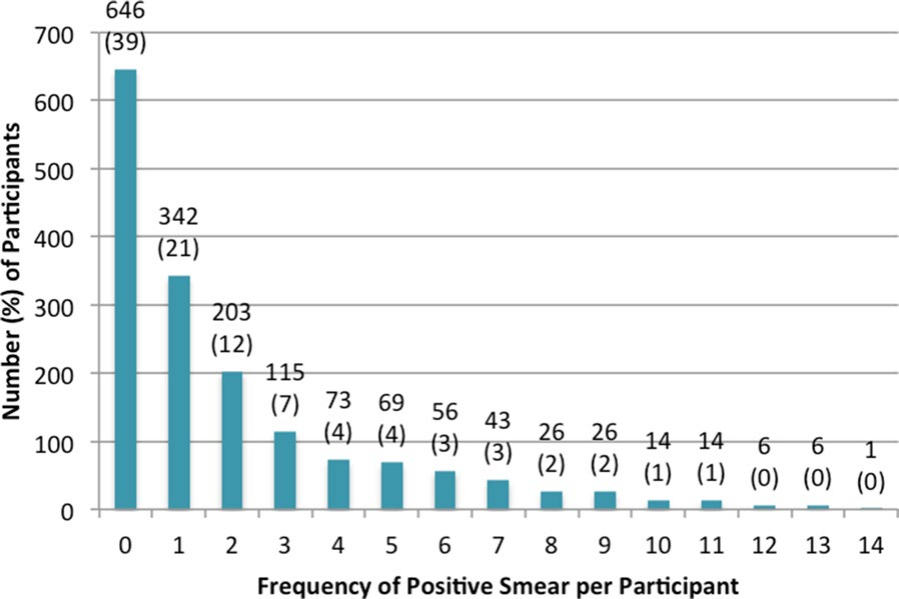
Frequency of positive smears for each participant during follow-up data value for each *bar* is number (%) of participants

Figure 1 shows the frequency of positive smear results (any level of parasitaemia) during the study period. 646 (39 %) had no positive smears; 994 (61 %) individuals had at least one positive smear of which 342 (21 %) had one positive result, 203 (12 %) had two, 115 (7 %) had three, and 334 (21 %) had >3 positive smear results during follow-up.

**Fig. 2:**
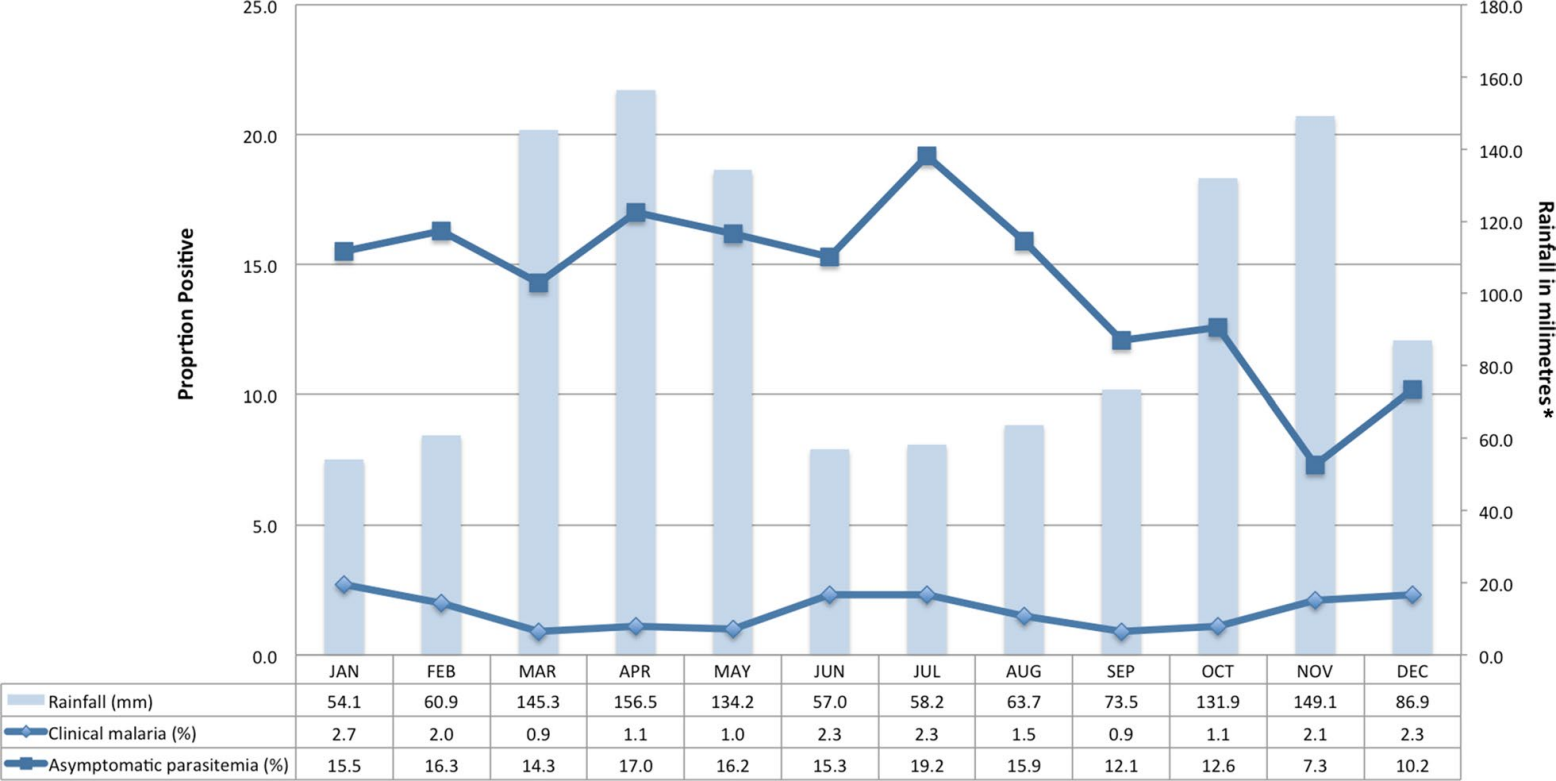
Malaria prevalence and average rainfall by month. *bar* rainfall* (mm), *lower line* clinical malaria, *upper line* asymptomatic parasitaemia. *mean historical monthly rainfall data for Rakai, Uganda during 1990–2009 was accessed on May 12, 2015 from The World Bank Group Climate Change Knowledge Portal: http://sdwebx.worldbank.org/climateportal/index.cfm?page=country_historical_climate&ThisRegion=Africa&ThisCCode=UGA

Figure 2 shows monthly prevalence of smear positive malaria outcomes during the study period. Seasonal rates generally followed the rains [8] and peaked during April to July, then decreased through November before increasing again.

There were speciation results for 3267 positive smear readings. *Plasmodium falciparum* was identified in 92 %, *P. vivax* in 6 %, and *P. malariae* in 6 % of smears. The predominance of *P. falciparum* was parallel in both clinical malaria and asymptomatic parasitemia groups (Table 4). There were 113 instances of multi-species infection, with 72 (2 %) cases identified with both *P. falciparum* and *P. vivax*; 40 (1 %) cases, with both *P. falciparum* and *P. malariae*; and one (0.03 %) case with both *P. vivax* and *P. malariae*.

**Table 4:** Speciation observed for positive smears (N = 3267)

Malaria species	Confirmed clinical malaria n (%)	Asymptomatic parasitaemia n (%)	Total n (%)
*Plasmodium falciparum*	220 (7)	2778 (93)	2998 (92)
*Plasmodium malariae*	3 (2)	193 (98)	196 (6)
*Plasmodium vivax*	10 (5)	176 (95)	186 (6)

Multiple species were identified for 113 positive smears, so the total count shows as higher than the number of positive slides

Fever (temperature of >37.5 °C) was identified at 467 (2.2 %) visits. 468 rapid diagnostic tests for malaria were performed in febrile participants and 347 (74.2 %) RDT samples were positive. Subsequent confirmation of these results by thick smear showed 242 (52 %) smear-positivity. There were concordant positive results for 218 (47 %) samples, concordant negative results for 113 (24 %) samples, and discordant results for 137 (29 %) samples: eight (2 %) samples were RDT-negative but smear-positive, and 129 (28 %) samples were RDT-positive, but smear-negative. The kappa statistic for level of agreement between RDT and smear result was 0.42 (95 % C.I. 0.35, 0.49).

The malaria attributable fraction among febrile episodes was consistent across age groups: 67 % among young children <5 years, 68 % in children 5–<10 years, 66 % in ages 10–<18 years, and 70 % in adults 18+ years. There was a notable difference between genders that was consistent across all age groups, with males demonstrating a higher fraction of fevers attributable to malaria. The MAF for fever among male participants was 76 %, whereas for females it was 58 %.

Prevalence of anaemia was determined for all febrile participants and results are presented in Table 5 only for participants <10 years since anaemia was very rare in older age groups. Among 336 children <10 with fever, 122 (36 %) showed anaemia of any severity level, of which 113 (93 %) had CCM. All 20 children identified with severe anaemia (hemoglobin < 8 g/dl) were CCM cases, and CCM cases were 3.86 times more likely to be anaemic compared to children without CCM (p < 0.0001). Younger children <5 years were 1.84 times more likely to be anaemic compared to older children 5–<10 years (p < 0.0001).

**Table 5:** Prevalence of anemia among children age 6 months–<10 years

	Severe anemia (<8 g/dl) n (%)	Moderate anemia (8–9.9 g/dl) n (%)	Mild anemia (10–10.9 g/dl) n (%)	Normal hemoglobin (11+ g/dl) n (%)	Count (N = 336)
Age group (years)					
0.5–<5	15 (8)	26 (15)	41 (23)	95 (54)	177 (53)
05–<10	5 (3)	21 (13)	14 (9)	119 (75)	159 (47)
Gender					
Male	14 (7)	25 (13)	30 (16)	119 (63)	188 (56)
Female	6 (4)	22 (15)	25 (17)	95 (64)	148 (44)
Cluster					
Kalisizo	7 (4)	29 (17)	31 (18)	103 (61)	170 (51)
Kabira	13 (8)	18 (11)	24 (14)	111 (67)	166 (49)
Confirmed clinical malaria status					
Yes	20 (8)	41 (16)	52 (20)	144 (56)	257 (76)
No	0 (0)	6 (8)	3 (4)	70 (89)	79 (24)
Total	20 (6)	47 (14)	55 (16)	214 (64)	336
Any anaemia	122 (36)				

Self-reported (or caregiver-reported for children) prevention methods used at baseline are summarized in Table 6. 192 (12 %) participants reported use of indoor residual spray (IRS) in the household, 656 (40 %) reported use of any mosquito bed net, of which 473 (29 %) reported the bed net was an insecticide-treated net (ITN), and 31 (2 %) reported using intermittent preventive treatment (IPT) for malaria. Caregivers reported using bed nets more frequently than other cohorts (p < 0.001).

**Table 6:** Self-reported malaria prevention methods at baseline

	Use of indoor residual spray n (%)	Use of any mosquito net n (%)	Use of insecticide- treated net n (%)	Use of intermittent preventive treatment n (%)	Count (N = 1640)
Cohort					
Caregiver	46 (12)	197 (50)	137 (35)	28 (7)	393 (24)
Adult	27 (10)	80 (29)	59 (22)	2 (0.7)	272 (17)
Child	119 (12)	379 (39)	277 (28)	1 (0.1)	975 (59)
Total	192 (12)	656 (40)	473 (29)	31 (2)	1640

## Discussion

This study describes the relatively high rates of malaria in rural Uganda that also has a high burden of HIV [7]. This is compatible with other studies in rural Uganda. A strength of this study is the longitudinal monthly assessments at the household level among a large number of individuals over a 1 year period and across a spectrum of age groups. Loss to follow-up was minimal and adherence was high. The study provides estimates of malaria incidence rather than prevalence alone, and during a time frame in rural Uganda not described by the periodic MIS rounds. Furthermore, the study also provides information on malaria prevention methods, and reports on prevalence of anaemia among febrile children. There are some important limitations of this study. Recruitment targeted households from two community clusters within Rakai district that provided convenient access to the study team due to their proximity to health centers and therefore these findings might not be generalizable to the entire district. The investigators did not ascertain HIV sero-status as part of the study; however, we these results are interpretable within the context of an area of known endemic HIV transmission and prevalence.

Findings from this study are consistent with reports from the Uganda MIS from 2009 to 2014/2015 (Rakai District is included in the Central 1 region), which show a downward trend for prevalence of malaria and anaemia in young children age 0–59 months. The current study from 2011/2012 is in between these MIS time points, and prevalence estimates for CCM in the youngest age cohort likewise fall in between (30 % in this study, versus 45 % from the MIS in 2009 and 13 % in 2014/2015). Prevalence of anaemia follows a similar trend, with 8 % of the youngest age cohort from the current study identified with severe anaemia (hemoglobin < 8 g/dl), compared to 11 and 4 % respectively from the MIS 2009 and 2014/2015; and 46 % of children <5 years from this study identified with any level of anaemia (hemoglobin < 11 g/dl) compared to 63 % form the MIS 2009. Reported use of malaria prevention strategies in the current study is also comparable to the Uganda MIS findings. Overall use of any mosquito bed net (40 %) and ITN (29 %) fall in between an apparent increasing trend reported in MIS (any bed net use in children was 32 % in 2009 and 68 % in 2014/2015, with use of ITN 22 % for children <5 years in 2009, and 59 % in 2014/2015).

As expected, the predominant species identified by microscopy was *P. falciparum*. The identification of *P. vivax* in East Africa is not unexpected, although this identification was done by smear and not by PCR. The presence of *P. vivax* in Duffy-negative individuals, as would be expected in East Africa, has been previously described in East Africa [14] and in the Amazon [15]. It is also possible that the specificity of microscopy was not optimal.

The discrepancy between RDT and smear positivity is also not unexpected but is higher than has been observed in other studies [1, 3]: for the 2 % of samples that were RDT negative but smear positive, it is possible that non-falciparum species accounted for the RDT “false negatives” since the sensitivity for non-falciparum species with this RDT is slightly lower. Indeed, for those eight (2 %) discordant samples of the samples we reported on, six (75 %) were positive for *P. falciparum*, one (12 %) was positive for *P. vivax*, and one (12 %) was positive for *P. malariae*. For the 28 % of RDTs that were positive but smear negative, it is possible that microscopy sensitivity was imperfect, or that the RDTs were picking up circulating antigen or parasites (gametocytes) that existed after patients had been recently treated for infection [16].

A higher degree of AP than CCM was observed in this study. If molecular methods had been employed in this study, we likely would have detected higher AP rates. Indeed, using sensitive molecular methods and targeting interventions to those with AP is important to eradication since it likely fuels the cycle of transmission. Community-wide drug treatment, indoor residual spraying, bed net distribution, and mosquito larviciding are all efforts that have been and could be guided by highly sensitive community screening and intervention efforts [17].

It is likely that children who had febrile illnesses who were RDT negative had infections other than malaria, and this has been increasingly described in the literature recently. In fact, recent studies have shown that with the decreasing incidence of malaria as evidenced by data in this study and the Uganda MIS 2009 and 2014/2015 [1, 3], etiologies of fever in younger children are much more likely to be due to viral illnesses than bacterial or parasitic infections (such as typhoid or malaria) [18].

## Conclusions

*Plasmodium falciparum* infection remains high in rural Rakai, Uganda. Increased malaria interventions should be implemented to reduce the burden of disease.
